# Angiography-based quantitative coronary contrast-flow ratio measurements correlate with myocardial ischemia assessed by stress MRI

**DOI:** 10.1007/s10554-020-01855-z

**Published:** 2020-05-04

**Authors:** Karsten Lenk, Valentin Schwarzbach, Marios Antoniadis, Maximilian Blum, Samira Zeynalova, Andreas Hagendorff, David Leistner, Ulf Landmesser, Daniel Lavall, Ulrich Laufs

**Affiliations:** 1 Department of Cardiology, University Hospital, Leipzig University, Leipzig, Germany; 2 Institute for Medical Informatics, Statistics and Epidemiology (IMISE), Leipzig University, Leipzig, Germany; 3 Department of Cardiology, Charité Berlin University Medicine, Campus Benjamin Franklin, Berlin, Germany

**Keywords:** Computational fluid dynamics, Coronary artery disease, Non-invasive imaging, Quantitative coronary angiography, Stress MRI

## Abstract

Contrast-flow quantitative flow ratio (cQFR) is a new technology for quantitative evaluation of coronary stenosis using computational fluid dynamics based on angiograms. The aim of this study was to assess the sensitivity and specificity of cQFR to detect myocardial ischemia using stress magnetic resonance imaging (MRI) as a reference standard. Patients who received stress MRI and coronary angiography were selected from the hospital database. Relevant ischemia on stress MRI was defined as a perfusion deficit in ≥ 2 of 16 segments. cQFR was quantitated based on 3-dimensional quantitative coronary angiography using QAngio XA3D1.1 software by two blinded and independent investigators. A cQFR of ≤ 0.80 was considered abnormal. Among 87 patients 230 vessels met the criteria for full analysis by cQFR (88%). In vascular territories with a significant perfusion deficit, cQFR was significantly lower compared to areas with normal perfusion (0.72 (0.62–0.78) vs. 0.96 (0.89–0.99); p < 0.001). The sensitivity of cQFR in detecting significant epicardial stenoses of coronary vessels with documented ischemia in stress MRI was 81% (68–90%), the specificity was 88% (82–92%). Diameter stenoses (DS) and area stenoses (AS) in vessels with positive stress MRI were significantly higher than in vessels without ischemia (DS 59.1% (49.4–68.4%) vs. 34.8% (27.1–46.1%) p < 0.001; AS 75.6% (63.0–85.2%) vs. 45.0% (30.8–63.6%), p < 0.001). The analysis reveals a high correlation between coronary stenosis measured by cQFR and ischemic areas detected by stress MRI. The data set the stage to plan randomized studies assessing cQFR measurements with regard to clinical outcomes.

## Introduction

It is state of the art to assess the hemodynamic relevance of intermediate coronary stenosis with fractional flow reserve (FFR) in the absence of evidence of ischemia [1]. Percutaneous coronary angioplasty (PCI) guided by FFR is related to a more favourable outcome compared to PCI based on angiography alone [2]. Although FFR is a widely accepted technique, in real world practice it is used only in 6.1% of cases [3]. This is most likely due to the costs of the pressure wire, the time needed for the procedure, the necessity of hyperemia for FFR evaluation with associated side effects and, most importantly, the potential complications of a coronary wire passage. In addition, FFR only allows assessing one coronary artery at a time, which is a significant disadvantage in multivessel disease. To overcome some of these shortcomings, a new method called contrast-flow quantitative flow ratio (cQFR) has been established using fluid dynamic equations on the basis of 3-dimensional quantitative coronary angiography (QCA) [4]. Whereas FFR depends on microcirculatory resistance [5], cQFR relies basically on fixed boundary conditions and high quality angiograms [6]. To better reflect individual blood flow the volumetric flow rate is derived from the ratio of 3D-QCA lumen volume to the contrast transport time, obtained by frame counting [6]. Given this, all three major vessels can be analysed one after another. Although this new technique has shown a high accuracy in determining the functional significance of coronary stenosis, using FFR as reference [7, 8], studies investigating its reliability in detecting relevant epicardial stenosis leading to ischemia in non-invasive stress testing are scarce [9, 10]. None-invasive stress tests have long been a gold standard for identifying relevant ischemia in patients with chronic coronary syndrome. Importantly, FFR was itself validated using a number of non-invasive modalities as reference standard for myocardial ischemia [11]. Stress MRI is one of the first guideline recommended choices approaching a patient with suspected chronic coronary syndrome [12].

The aim of this study was therefore to analyse the sensitivity and specificity of cQFR in detecting ischemia assessed by stress MRI.

## Methods

Patients were selected from the hospital database who received stress MRI and coronary angiography. Exclusion criteria were insufficient quality of angiograms or stress MRI, patients with more than one chronic total occlusion (CTO) or patients post coronary artery bypass grafting. Exclusion criteria for the analyses of coronary vessels included the absence of 2 angiographic projections more than 25° apart, coronary artery occlusions, foreshortening, ostial lesions and insufficient image quality.

### Stress MRI

Stress MRI was performed using a 3 T System (Achieva, Philips Medical System, Best, The Netherlands) with a 32-channel phased-array surface coil with dS anterior and posterior coil. Beta blocker, caffeine or methylxanthines were stopped for at least 24 h prior to the examination. Non-invasive monitoring of heart rate, blood pressure, and oxygen saturation was performed during MRI.

#### Data acquisition

Cine images were acquired covering the entire left ventricle in contiguous short axis and longs axis using a steady-state free-precession sequence (slice thickness 8 mm, no interslice gap). After 30 s 400 µg bolus injection of regadenoson (GE, Norway), myocardial first pass of 4.5 mmol Gadolinium-based contrast agent (Gadobutrol, Bayer, Germany) was visualized in three short axes. A steady-state free- precession sequence was used for perfusion imaging (slice thickness 8 mm; interslice gap 9 mm) [13].

#### MRI analysis

A blinded and experienced reader performed the analysis of all stress MRI images. Regadenoson first pass perfusion images were analysed semi-quantitatively. The myocardium was divided into 16 segments (excluding the apical cap of the 17 segments AHA model) [14, 15]. Ischemia was assessed visually and was defined as dark myocardium for ≥ 3 frames, involving ≥ 1/3 of the left ventricular wall thickness and at least 50% of the circumferential extent of the myocardial segment. Perfusion defects were considered in late gadolinium enhancement-negative myocardial segments only, i.e. in the viable myocardium. The segments were then assigned to the respective perfusion territory depending on coronary dominance [14, 15]. A relevant perfusion deficit was defined when two or more segments (> 10% of myocardium) were involved.

### Measurement of 3D-QCA, cQFR and fQFR

Two blinded investigators independently performed all QFR analyses in parallel for all three main coronary vessels. For each coronary artery, two appropriate projections separated at least 25 degrees with a minimum of vessel overlap and foreshortening were chosen. Using the enddiastolic phase in both projections, vessel wall contours were automatically detected and manually adjusted to generate the reconstruction of a 3D-model of the coronary artery. Angiographic stenosis severity was determined by percent DS and percent AS from 3D-QCA. By utilizing the Thrombolysis In Myocardial Infarction (TIMI) frame counting method, the contrast flow velocity was calculated leading to a cQFR value as reported [8]. 3D-QCA and cQFR were obtained using the QAngio-XA 3D software (version 1.1, provided by Medis, Leiden, The Netherlands) [6]. Furthermore, the fixed flow QFR (fQFR) was computed using an empiric hyperemic flow velocity of 0.35 m/s [6, 7]. Vessels without obvious lesions where measured in their mid-part. Dominant obtuse marginal branches were measured in patients with small circumflex arteries. As recommended by previous publications, a cQFR or fQFR of ≤ 0.80 was regarded pathologic [7, 16].

### Statistical analyses

Normally distributed continuous variables were analysed using the Student’s *t*-test. Data are presented as mean ± standard deviation. In case variables were not normally distributed the Mann–Whitney U test was used and data are presented using the median together with a 25–75% interquartile range (IQR). The diagnostic performance of cQFR for predicting presence of myocardial ischemia in a vascular territory diagnosed by stress MRI was assessed by sensitivity, specificity, positive predictive value and negative predictive value. To characterize the diagnostic performance of cQFR for predicting stress MRI positive vessel territories sensitivity, specificity, positive predictive value, negative predictive values and diagnostic accuracy were calculated and presented with their corresponding 95% confidence intervals. To visualize the diagnostic accuracy of cQFR and fQFR for identifying stress MRI positive vessel territories as a reference standard receiver-operating characteristic (ROC) curves were constructed using area under the curves (AUCs) with 95% confidence intervals. To evaluate the effects of cQFR and clinical parameters on the presence of a significant myocardial perfusion deficit, a binomial logistic regression was implemented. An interobserver comparison was accomplished using pearsons correlation coefficient. All statistical analyses were performed with the SPSS software package (IBM Corp. Released 2018. IBM SPSS Statistics for Windows, Version 24.0. IBM Corp.e, Chicago, IL, USA). A two-sided p-value of 0.05 was considered statistically significant.

## Results

Between 2017 and 2019, in total 87 patients were identified who fulfilled all inclusion and exclusion criteria. Out of 261 potential vessels, 230 met the quality criteria for analysis. 31 (12%) coronary arteries had to be excluded due to absence of angiographic projection angles more than 25° apart (n = 13), coronary artery occlusion (n = 4), foreshortening (n = 3), ostial lesions (n = 1) and insufficient image quality (n = 10). It has to be noted that the angiographies were retrospectively analyzed and were therefore not performed using the recommended projections for optimal vessel analysis with cQFR.

### Patient characteristics

The patient baseline and procedural characteristics are shown in Table 1. The majority of patients were male (76%), mean age was 65 ± 10 years, mean ejection fraction was 55 ± 14%. The cardiovascular risk factor profile and the medication are typical for a coronary artery disease patient cohort.

**Table 1:** Patient characteristics

	n = 87
Age (y)	65 ± 10
Male sex (%)	76
Hight (cm)	172 ± 9
Weight (kg)	78 ± 13
Body mass index	27 ± 4
Systolic blood pressure (mmHg)	143 ± 21
Diastolic blood pressure (mmHg)	84 ± 14
Echocardiographic Left ventricular ejection fraction (%)	55 ± 14
**Cardiovascular risk factors**	**n (%)**
Diabetes	28 (33)
Insulin dependent	4 (5)
Hypertension	69 (81)
Smoking	37 (44)
Previous smoking	14 (17)
Hyperlipidemia	40 (47)
Family history of Coronary Artery Disease	16 (19)
**Medical history**	
Previous myocardial infarction	24 (28)
STEMI	20 (21)
Atrial fibrillation	6 (7)
Previous cerebral insult	1 (1)
COPD	7 (8)
Asthma	4 (5)
PAD	5 (6)
**Clinical presentation**	
Silent ischemia	21 (24)
Stable Angina pectoris	33 (38)
Evaluation of non-culprit lesion after myocardial infarction	13 (15)
Dyspnoea	18 (21)
Atypical Angina pectoris	2 (2)
**Vessel characteristics**	
No coronary artery disease	21 (24)
Single vessel disease	13 (15)
Two vessel disease	29 (33)
Three vessel disease	24 (28)
Tandem stenosis	54/261 (21)
Diffuse disease	44/261 (17)
Mean reference vessel diameter (3D-QCA; mm)	2,5 ± 0.7
Lesion length (3D-QCA; mm)	17 ± 10
**Medication**	
ASA	45 (53)
Clopidogrel/Ticagrelor	12 (14)
Betablocker	55 (65)
ACE-inhibitor	34 (40)
ARB	26 (31)
Statin	47 (55)
Nitrates	4 (5)
Ranolazine	2 (2)
Ivabradine	1 (1)
Calcium channel blocker	18 (21)
Oral anticoagulation	13 (15)
Glycosides	4 (5)

Values are given as median ± standard deviation

*STEMI-ST* segment elevation myocardial infarction, *COPD* chronic obstructive pulmonary disease, *PAD* peripheral artery disease, *ASA* acetyl salicylic acid, *ACE* angiotensin-converting enzyme, *ARB* angiotensin-II-receptor blocker

### Stress MRI

As shown in Table 2, the mean ejection fraction measured by MRI was 57 ± 13%, the left ventricular enddiastolic diameter 51 ± 8 mm. After administration of regadenoson, the heart rate increased by 22 ± 9 beats per minute. In the stress MRI analyses, 59% (n = 51) of all patients had a relevant ischemic burden (> 10%). The vascular territories of the left anterior descending artery (LAD), right coronary artery (RCA) and circumflex artery (LCX) showed significant perfusion deficits in 42% (n = 37), 23% (n = 20), and 9% (n = 8) of analysed vessels, respectively.

**Table 2:** MRI characteristics

	n = 87
LVEDD (mm)	51 ± 8
LVEDV (ml)	148 ± 58
LVESD (mm)	37 ± 10
LVESV (ml)	71 ± 53
LV SV (ml)	79 ± 17
Left ventricular ejection fraction (%)	57 ± 13
Heart rate begin (/min)	69 ± 9
Heart rate after Regadenoson (/min)	92 ± 9
Delta Heart rate	22 ± 9
Segments per patient with perfusion deficit	2 (0–4)
Patients with relevant ischemia	51 (59%)

Values are mean ± standard deviation, median and interquartile range or n (%)

*LVEDD* left ventricular enddiastolic diameter, *LVEDV* left ventricular enddiastolic volume, *LVESD* left ventricular endsystolic diameter, *LVESV* left ventricular endsystolic volume, *LVSV* left ventricular stroke volume

### Vessel characteristics

**Fig. 1:**
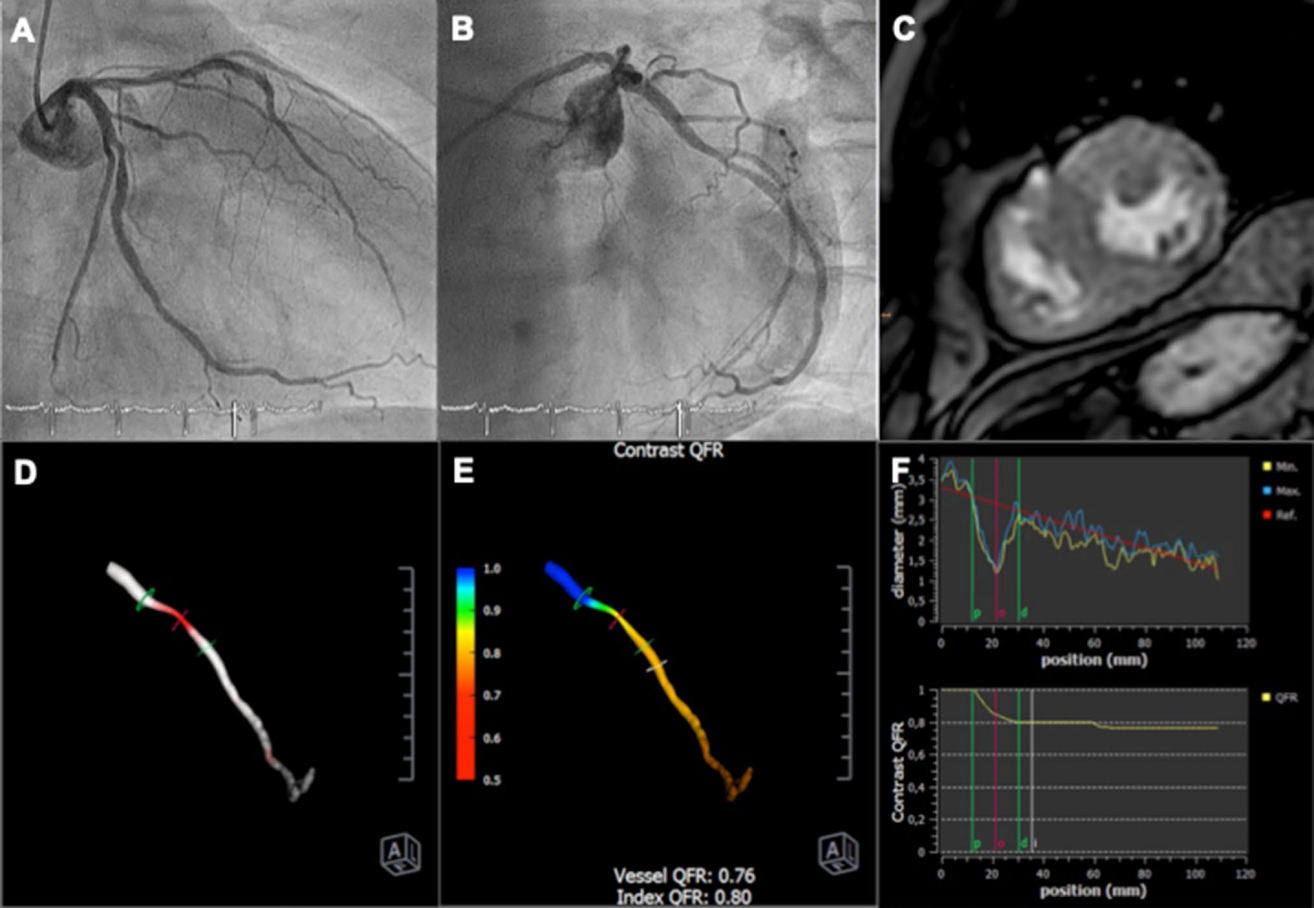
Representative examples. **a** Right anterior oblique (RAO) 30° projection of the left coronary artery. **b**, Coronary angiogram of the left coronary artery in left anterior oblique (LAO) 60° angulation. **c**, Stress MRI showing a perfusion deficit in the midventricular inferolateral segment of the left ventricle. **d**, 3D-Reconstruction of the dominant 2nd marginal branch in LAO 28° and 24° caudal projection. **e**, 3D Reconstruction and visualisation of the dominant 2nd marginal branch with QFR. Vessel QFR characterises the whole vessel and index QFR pointes out the QFR at the white marker. **f**, Lumen dimensions showing minimal, maximal and reference diameters (above) and virtual cQFR pullback (below) with p representing the proximal lesion marker, d the distal lesion marker and o the lesion centre

**Fig. 2:**
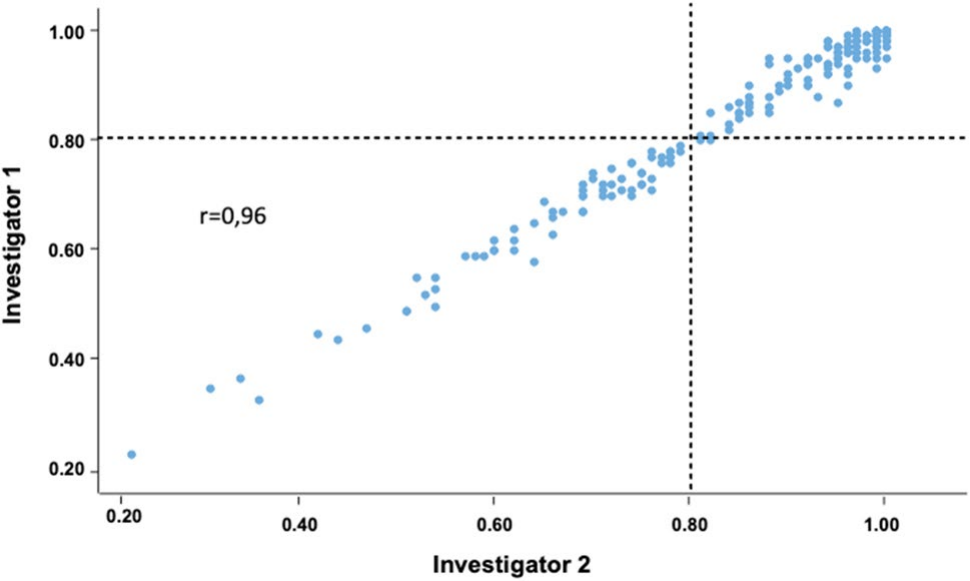
Correlation of all measured cQFR values between the two blinded investigators

The vessel characteristics are displayed in Table 3. Of all analysed vessels, the mean cQFR was 0.94 (0.76–0.99) and the mean fQFR was 0.95 (0.78–0.99). The percentage diameter stenoses (DS) was 42.3% (28.4–55.5%), the percentage area stenoses (AS) was 52.8% (32.7–72.0%) and the minimal lumen diameter (MLD) was 1.4 ± 0.6 mm. Figure 1 shows a representative example of a patient with a perfusion deficit on stress MRI and an abnormal cQFR. Figure 2 depicts the correlation of the cQFR measurements between the two independent investigators.

**Table 3:** Vessel characteristics

			MRI positive		MRI negative		p-value	Total
cQFR	LAD	n = 35	0.70 (0.62–0.76)	n = 45	0.93 (0.84–0.97)		< 0.001	
	RCA	n = 17	0.75 (0.63–0.99)	n = 62	0.97 (0.88–1.00)		< 0,001	
	LCX	n = 6	0.77 (0.75–0.78)	n = 65	0.98 (0.94–1.00)		< 0,001	
	All	n = 58	0.72 (0.62–0.78)	n = 172	0.96 (0.89–0.99)	n = 230	< 0,001	0.94 (0.76–0.99)
fQFR	LAD	n = 35	0.71 (0.62–0.80)	n = 45	0.93 (0.80–0.97)		< 0,001	
	RCA	n = 17	0.76 (0.58–0.99)	n = 62	0.97 (0.90–1.00)		< 0,001	
	LCX	n = 6	0.79 (0.72–0.85)	n = 65	0.99 (0.95–1.00)		< 0,001	
	All	n = 58	0.73 (0.62–0.83)	n = 172	0.97 (0.89–0.99)	n = 230	< 0,001	0.95 (0.78–0.99)
DS	LAD	n = 35	58.4 (50.2–68.5)	n = 45	36.8 (29.9–49.2)		< 0,001	
	RCA	n = 17	56.9 (28.6–66.4)	n = 62	34.8 (25.5–44.6)		< 0,001	
	LCX	n = 6	64.4 (52.5–70.8)	n = 65	33.9 (26.3–41.6)		< 0,001	
	All	n = 58	59.1 (49.4–68.4)	n = 172	34.8 (27.1–46.1)	n = 230	< 0,001	42.3 (28,4–55,5)
AS	LAD	n = 35	75.0 (65.0–85.0)	n = 45	44.1 (31.7–67.8)		< 0,001	
	RCA	n = 17	75.3 (34.1–84.2)	n = 62	47.2 (29.7–61.5)		< 0,001	
	LCX	n = 6	84.6 (72.8–87.9)	n = 65	43.3 (30.8–56.4)		< 0,001	
	All	n = 58	75.6 (63.0–85.2)	n = 172	45.0 (30.8–63.6)	n = 230	< 0,001	52,8 (32,7–72,0)
MLD	LAD	n = 35	0.9 ± 0.3	n = 45	1.3 ± 0.4		< 0,001	
	RCA	n = 17	1.3 ± 0.6	n = 62	1.9 ± 0.6		< 0,001	
	LCX	n = 6	1.1 ± 0.5	n = 65	1.4 ± 0.5		< 0,001	
	All	n = 58	1.0 ± 0.5	n = 172	1.5 ± 0.6	n = 230	< 0,001	1,4 ± 0.6

Vessel characteristics depending on stress MRI positive or MRI negative results, Values are given as median and interquartile range, or mean ± standard deviation

*DS* %diameter stenoses, *AS* %area stenoses, *MLD* minimal luminal diameter

### cQFR, fQFR, anatomical indices and baseline parameters in relation to stress MRI results

**Fig. 3:**
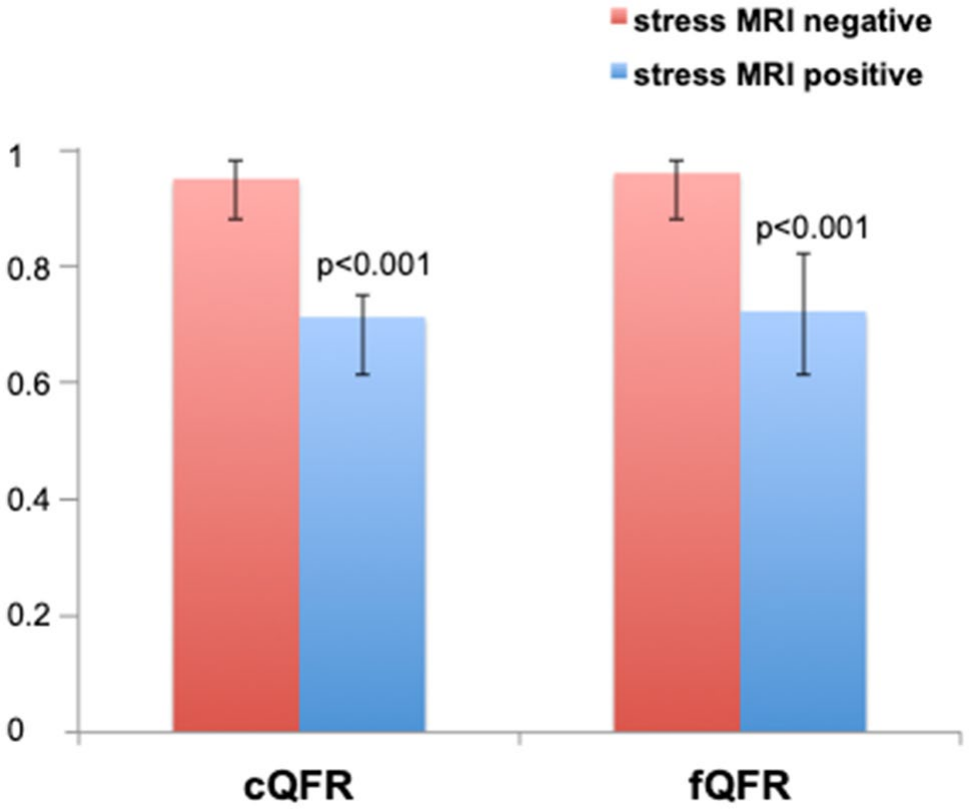
Contrast-flow quantitative flow ratio (cQFR) and fixed flow QFR (fQFR) in relation to relevant ischemia on stress stress magnetic resonance imaging (stress MRI) defined as a perfusion deficit in ≥ 2 of 16 segments depicted as median and interquartile range

In vascular territories with a significant perfusion deficit, values for cQFR and fQFR were significantly lower compared to vascular territories with normal perfusion (0.72 (0.62–0.78) vs. 0.96 (0.89–0.99) p < 0.001; 0.73 (0.62–0.83) vs. 0.97 (0.89–0.99) p < 0.001 respectively; Fig. 3). Mean area stenosis was 75.6% (63.0–85.2%) vs. 45.0% (30.8–63.6%); p < 0.001 and mean diameter stenosis was 59.1% (49.4–68.4%) vs. 34.8% (27.1–46.1%); p < 0.001 in vessels with a relevant perfusion deficit. Minimal lumen diameter was significantly lower in coronary arteries with corresponding stress MRI positive results (1.0 ± 0.5 vs. 1.5 ± 0.6 mm, p < 0.001).

**Fig. 4:**
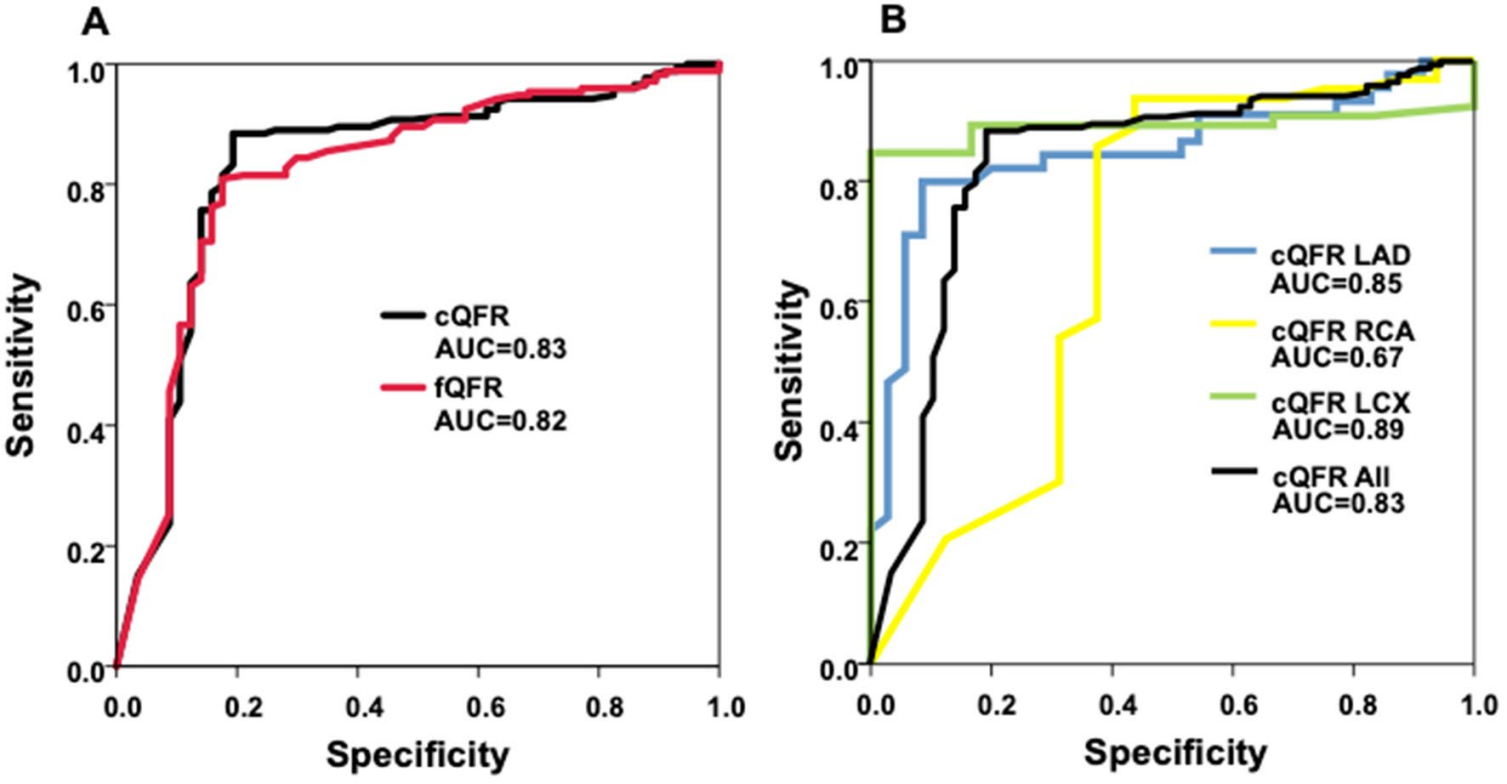
**a**, ROC-curves of cQFR and fQFR for all measured vessles. **b**, ROC-curves of cQFR values of LAD, RCA and LCX compared to cQFR of all vessels

**Fig. 5:**
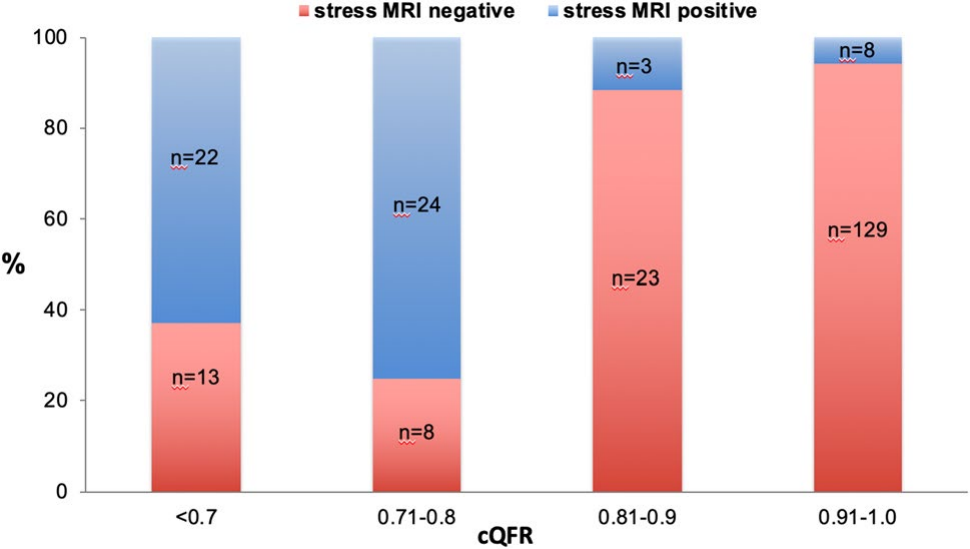
Distribution of stress MRI positive and negative results within four different cQFR strata (< 0,7; 0.71–0.8; 0.81–0.9; 0.91–1.0). Each column represents the percentage of stress MRI positive and negative results within the predefined cQFR range

The diagnostic performance of cQFR in relation to stress MRI is depicted in Table 4 and in Fig. 4. The area under the receiver-operating characteristic curve (AUC) for cQFR, fQFR and 3D-QCA DS was 0.83, 0.82 and 0.81 respectively for all lesions. Looking into the different vessel territories, the AUC for cQFR of all RCA lesions was 0.69 compared to 0.85 and 0.89 for all LAD and LCX lesions respectively (Fig. 4b). In Fig. 5 the percentages of cQFR-results in different cQFR-strata for stress MRI positive and for stress MRI negative territories are visualized.

**Table 4:** Diagnostic performances

	Sensitivity	Specificity	PPV	NPV	Diagnostic accuracy
LAD	91% (71; 98)	80% (65; 90)	78% (62; 89)	92% (79; 98)	85% (81; 90)
RCA	56% (30; 80)	92% (82; 97)	64% (35; 87)	89% (79; 96)	85% (75; 92)
LCX	83% (36; 100)	89% (79; 96)	42% (15; 72)	98% (91; 100)	89% (79; 95)
All	81% (68; 90)	88% (82; 92)	69% (56; 79)	93% (88; 97)	86% (81; 90)

Parameters of diagnostic performance of the cQFR in the three main coronary vessles given in percent and 95% confidence interval in brackets

*LAD* left anterior descending artery, *LCX* circumflex artery, *RCA* right coronary artery, *PPV* positive predictive value, *NPV* negative predictive value

The multivariate analysis showed a very high discriminative power of cQFR for detecting stress MRI negative perfusion areas compared to other baseline parameters (Table 5).

**Table 5:** Multivariate analyses

	RR	95% CI	p-value
Gender	1.0	(0.3; 2.8)	0.894
Age	1.0	(1.0; 1.1)	0.283
HF	1.0	(1.0; 1.0)	0.762
BP	1.0	(1.0; 1.0)	0.866
Diabetes	1.2	(0.9; 1.5)	0.277
Smoker	0.9	(0.5; 1.6)	0.685
Hyperlipidemia	1.7	(0.7; 4.3)	0.241
Heart attack	0.8	(0.4; 1.8)	0.615
LVEF	1.0	(1.0; 1.0)	0.755
QFR ≤ 0.8	26.9	(11.4; 63.5)	< 0.001

Multivariate analysis of various baseline parameters for the detection of ischemia with stress MRI in a vascular territory

*HLP* hyperlipoproteinemia, *MI* myocardial infarction, *HR* heart rate, *BP* blood pressure, *LVEF* left ventricular ejection fraction

## Discussion

The study analysed the performance of the contrast-flow quantitative flow ratio, a novel angiography-based technique to quantitate the hemodynamic relevance of coronary lesions. The main novel finding of the study is that cQFR correlates with significant perfusion-deficits documented by stress MRI with a sensitivity of 81%, a specificity of 88% and a diagnostic accuracy of 86%.

The prognosis of patients with coronary artery disease is closely related to the functional significance of the stenosis [17]. Stress MRI is an established and recommended non-invasive approach for the detection of myocardial ischemia [18]. Recent guidelines define a perfusion deficit ≥ 10% as prognostically relevant [1]. This is equivalent to ≥ 2 out of 16 segments with stress induced perfusion deficits in MRI [17]. Comparing perfusion on stress MRI to FFR-positive lesions, rates of sensitivity range between 88 and 92% and specificity between 56.7–94% in various studies [19–21]. This close relationship underlines the diagnostic value of both methods. The recently published MR-INFORM trial demonstrated the use of myocardial- perfusion cardiovascular MRI in guiding initial management of patient care was noninferior to the use of invasive coronary angiography combined with FFR with respect to the primary outcome of major adverse cardiac events at 1 year [22]. Until now only one study has investigated the association between stress MRI and cQFR [9]. Interestingly, there is an obvious difference between the two studies regarding sensitivity and specificity. Several reasons might be responsible. One of the main reasons is the burden of disease which is reflected by the different QFR-values. Our study had a mean QFR of 0.94, which is in contrast to the study of Sejr-Hansen and colleagues with a mean QFR of 0.84 [9]. Another reason might be the selection process of patients for the study. The initial step in the study of Sejr-Hansen was a coronary computed tomography angiography (CCTA). Out of these CCTA-positive patients only 25% had perfusion defects on myocardial perfusion scintigraphy or stress MRI. Compared to our study with an ischemic burden in 59% of patients this may lead to different results. It is possible that patients selected by CCTA have a higher burden of “visible” disease which does not lead to perfusion deficits.

FFR-measurement represents the method of choice in evaluating coronary stenoses of epicardial vessels because of the documented prognostic relevance [1, 23]. Recent studies report a high correlation of QFR with FFR measurements [8, 16]. Importantly, to date no randomised trial with clinical outcomes has proven non-inferiority of QFR compared to FFR but studies are ongoing (i.e. FAVOR III Europe-Japan; NCT03729739). The FAVOR II China study compared FFR with cQFR using optimised projections and reported good agreement between cQFR and FFR (mean difference: − 0.01 ± 0.06; p = 0.006) [16]. The FAVOR II study performed in Europe and Japan confirmed that the precision (absolute difference of QFR-FFR) for QFR with FFR as a reference was not different across strata of FFR values [8]. Furthermore, Stahli et al. found that the diagnostic accuracy of QFR was superior compared to the wave free index distal to aortic coronary pressure (Pd/Pa) using FFR as gold standard [24]. Comparisons between QFR and single-photon emission computed tomography myocardial perfusion imaging (SPECT MPI) revealed a good correlation [10]. Our study extends these data to the important parameter of myocardial ischemia as quantitated by stress MRI. In addition, the study demonstrates the robustness of the method for retrospective analysis of routine angiograms performed without using ideal "QFR-projections". This information is very important for all researchers that plan to perform retrospective analyses of coronary angiograms. From this perspective the very high inter-observer correlation of the QFR quantifications between the independent and blinded investigators in our study is important and in agreement with the literature [25].

Our data reveal that cQFR is able to detect relevant coronary stenoses in the vast majority of patients with a positive stress MRI and that these correlate with the respective territory of ischemia. Interestingly, the study reveals differences between the three main coronary arteries. The sensitivity of cQFR in relation to stress MRI was reduced for the RCA compared to LAD or LCX-lesions. This might be due to suboptimal angulation of angiograms, as these were not optimised for QFR-analyses. The two standard projections of the RCA differ from recommended optimal QFR-projections and offer reduced possibilities for compensation compared to the s standard left coronary artery-projections. A second explanation could be the anatomical course of the RCA with two rectangular deviations compared to the run of the left coronary arteries. To our knowledge differences in QFR performance between the three coronary main vessels have not been studied elsewhere and we cannot fully exclude a play of chance. However, the overall performance of the method in these routine angiograms, which were not optimised regarding preferred projection conditions, was good. Apart from these considerations, a certain difference between QFR and stress MRI is expected as MRI depicts the perfusion in a certain area whereas fractional flow reserve evaluates an isolated epicardial vessel. Entities such as small vessel disease or other dysfunctions of the microvasculature will not be addressed by these methods. Therefore, it is feasible that false negative results might be related to the status of the coronary microcirculation. On the other hand, stress MRI studies suggest that perfusion deficits of ≥ 10% correspond to FFR values below 0.67 [26]. Therefore, vessels with QFR-values of 0.7–0.8 may show a higher rate of mismatch compared to patients with QFR-values between 0.7–0.6 or lower. However, this suggestion was not confirmed in our study, as demonstrated in Fig. 5.

The multivariate analyses underline that clinical parameters are not helpful in deciding about the hemodynamic relevance of the disease, whereas cQFR provides good discrimination of vessels with or without associated ischemia.

## Limitations

Due to the retrospective nature of the study a selection bias cannot be excluded. Since patients with non-significant ischemia on stress MRI are less likely to undergo angiography, the study is not designed to assess the rate of false-positive cQFR results. Angiography was not performed with the aim to calculate cQFR. The use of routine projections rather than those recommended for cQFR may lead to a potential underestimation of the predictive power of cQFR.

Another potential limitation is that the correct assignment of the coronary artery to the perfusion of specific myocardial segments can be uncertain in some individuals [15].

The potential of cQFR relates to the absent risk of wire placement and contrast- or vasodilating agents, the option of off-line (and, as demonstrated in this study, retrospective) quantification and the unique possibility to assess all three coronary arteries. Our analysis reveals a high correlation between hemodynamically relevant coronary stenosis measured by cQFR and ischemic areas detected by stress MRI. On the basis of these promising results, randomized studies can be designed and are mandatory to verify the prognostic significance of cQFR measurements.

## Abbreviations

AS: Area stenoses
cQFR: Contrast-flow quantitative flow ratio
CTO: Chronic total occlusion
DS: Diameter stenoses
FFR: Fractional flow reserve
fQFR: Fixed flow quantitative flow ratio
IQR: Interquartile range
LAD: Left anterior descending artery
LCX: Circumflex artery
MLD: Minimal lumen diameter
MRI: Magnetic resonance imaging
PCI: Percutaneous coronary angioplasty
QCA: Quantitative coronary angiography
RCA: Right coronary artery
TIMI: Thrombolysis in Myocardial Infarction
